# Increased Prevalence of *NLRP3* Q703K Variant Among Patients With Autoinflammatory Diseases: An International Multicentric Study

**DOI:** 10.3389/fimmu.2020.00877

**Published:** 2020-05-14

**Authors:** Katerina Theodoropoulou, Helmut Wittkowski, Nathalie Busso, Annette Von Scheven-Gête, Isabelle Moix, Federica Vanoni, Veronique Hengten, Gerd Horneff, Johannes-Peter Haas, Nadine Fischer, Katharina Palm-Beden, Rainer Berendes, Georg Heubner, Annette Jansson, Elke Lainka, Annette Leimgruber, Michael Morris, Dirk Foell, Michaël Hofer

**Affiliations:** ^1^Pediatric Rheumatology Unit of Western Switzerland, Pediatric Department, Lausanne University Hospital (CHUV), Lausanne, Switzerland; ^2^Pediatric Department, Geneva University Hospital (HUG), Geneva, Switzerland; ^3^Department of Biochemistry, University of Lausanne (UNIL), Lausanne, Switzerland; ^4^Department of Pediatric Rheumatology and Immunology, University Children's Hospital, Muenster, Germany; ^5^Service of Rheumatology, DAL, Lausanne University Hospital (CHUV), University of Lausanne, Lausanne, Switzerland; ^6^Department of Genetics, SYNLAB, Lausanne, Switzerland; ^7^Istituto Pediatrico Della Svizzera Italiana, Bellinzona, Switzerland; ^8^Department of General Pediatrics, French Reference Center for Autoinflammatory Diseases (CEREMAIA), Versailles Hospital, Versailles, France; ^9^Asklepios Children's Hospital, St. Augustin, Germany; ^10^German Center for Pediatric and Adolescent Rheumatology, Garmisch-Partenkirchen, Germany; ^11^Department of Pediatric Rheumatology, St. Josef-Stift Sendenhorst, Sendenhorst, Germany; ^12^Departement of Pediatric Rheumatology, St. Marien-Childrens-Hospital Landshut, Landshut, Germany; ^13^Departement of Pediatrics, Städtisches Klinikum Dresden, Dresden, Germany; ^14^Department of Pediatrics, Dr. von Hauner Children's Hospital, University Hospital LMU Munich, Munich, Germany; ^15^Division of Pediatric Rheumatology, University Hospital Essen, Essen, Germany; ^16^Service of Immunology and Allergology, Lausanne University Hospital (CHUV), University of Lausanne, Lausanne, Switzerland

**Keywords:** Q703K, NLRP3, PFAPA, CAPS, autoinflammation, autoinflammatory diseases, recurrent fever

## Abstract

**Background:** The *NLRP3* inflammasome has been recognized as one of the key components of innate immunity. Gain-of-function mutations in the exon 3 of *NLRP3* gene have been implicated in inflammatory diseases suggesting the presence of functionally important sites in this region. Q703K (c.2107C>A, p.Gln703Lys, also known in the literature as Q705K) is a common variant of *NLRP3*, that has been considered to be both clinically unremarkable or disease-causing with a reduced penetrance.

**Objectives:** We aimed to investigate the potential genetic impact of the *NLRP3* variant Q703K in patients with recurrent fever presenting with two autoinflammatory diseases: PFAPA (periodic fever, aphthous stomatitis, pharyngitis and cervical adenitis) and CAPS (cryopyrin-associated periodic syndrome), as well as with undefined autoinflammatory disease (uAID).

**Methods:** This is an international multicentric observational retrospective study characterizing the clinical phenotype of patients presenting with recurrent fever suspected to be of auto-inflammatory origin and where the Q703K *NLRP3* variant was found. Monocytes of parents of 6 Q703K+ PFAPA patients were studied and levels of pro-inflammatory cytokines produced by monocytes of Q703K+ and Q703K- parents have been compared by ELISA.

**Results:** We report 42 patients with the Q703K *NLRP3* genetic variant: 21 were PFAPA patients, 6 had a CAPS phenotype, and 15 had an uAID. The phenotypes of PFAPA, CAPS and uAID were quite similar between Q703K positive and negative patients with the exception of increased prevalence of pharyngitis in the Q703K positive CAPS population compared to the negative one. The *in vitro* production of IL-1β was not significantly different between Q703K+ and Q703K- monocytes from asymptomatic parents.

**Conclusion:** The evidence we report in our study shows an increased prevalence of *NLRP3* Q703K in patients with autoinflammatory diseases, suggesting an association between the Q703K variant and the risk of PFAPA, CAPS and uAID syndromes. However, we did not show a functional effect of this mutation on the inflammasome basal activity.

## Introduction

Autoinflammatory diseases (AIDs) are clinically defined by repeating seemingly unprovoked attacks of multisystemic inflammation without underlying infection or autoantibody formation (1, 2). Monogenic origins have been established for some AIDs, including Familial Mediterranean Fever (FMF), Mevalonate Kinase Deficiency (MKD) also known as Hyperimmunoglobulinemia D and periodic fever syndrome (HIDS), TNF Receptor 1-Associated Syndrome (TRAPS) and Cryopyrin-Associated Periodic Syndrome (CAPS) (3). The pathophysiology of all these diseases is characterized by immune dysregulation due to enhanced IL-1β maturation, caused by direct or indirect inflammasome activation.

PFAPA (periodic fever, aphthous stomatitis, pharyngitis and cervical adenitis) syndrome is considered to be an AID without defined genetic origin (2). It represents the most common cause of recurrent fever in children in European populations, and an annual incidence of 2.3 cases for 10'000 children per year has been recently reported (4, 5). It is characterized by regularly recurring episodes of high fever, accompanied by at least one of the three cardinal symptoms, including pharyngitis, cervical adenitis and aphthous stomatitis. Patients are relatively asymptomatic between attacks and show normal growth and development (6, 7). The diagnosis is based on clinical criteria (7, 8) and the exclusion of other causes of recurrent fever, such as infectious, autoimmune and malignant diseases. Generally the onset of disease dates before the age of 5 years and these febrile episodes usually resolve spontaneously before adulthood (9). The rapid response of the fever attacks to a single dose of corticosteroid, the absence of an infectious or autoimmune cause, and the dysregulation of interleukin (IL-)1β secretion during fever flares supports the hypothesis that PFAPA syndrome is an autoinflammatory disease (10, 11).

The NLRP3 inflammasome (formerly known as NALP3 or cryopyrin) has been recognized as one of the key components of innate immunity by sensing microbial ligands, endogenous danger signals and crystalline substances in the cytosol. Upon activation, the sensor protein NLRP3 assembles with the adaptor protein ASC and pro-caspase-1 to form the NLRP3 inflammasome (12). This interaction leads to the activation of caspase-1, which proteolytically processes pro-IL-1β and pro-IL-18 to form, respectively, active IL-1β and IL-18 (13). Gain-of-function mutations in exon 3 of the *NLRP3* gene have been implicated in hereditary auto-inflammatory diseases, grouped under Cryopyrin-associated periodic syndrome (CAPS), suggesting the presence of functionally-important sites in this region (14).

Interestingly, Q703K (rs35829419, c.2107C>A, p.Gln703Lys, also known in the literature as Q705K) is a rather common *NLRP3* variant in exon 3, of unclear pathogenic significance, that has variously been considered to be clinically unremarkable and a disease causing with reduced penetrance. However, it has been found to be associated with PFAPA syndrome, CAPS (15, 16) and other inflammatory diseases (17, 18). Moreover, its functional role in inflammasome activation is ambiguous, as it was demonstrated to lead to increased IL-1β secretion *in vitro* (19), but not in healthy and CAPS carriers (12, 20).

These genetic observations, together with the association of dysregulated levels of IL-1β, raised the question on the role of the Q703K *NLRP3* variant in auto-inflammation. We therefore screened children with recurrent fever suspected to be of autoinflammatory origin followed in JIR cohort and AID registry, for variants of genes involved in monogenic AID (genes: *NLRP3, MEFV, TNFRSF1A, MVK*) in order to investigate the prevalence of the Q703K variant in patients with autoinflammatory diseases and we characterized the phenotype in mutation-positive patients. Furthermore, we compared inflammasome basal activity in 6 healthy Q703K carriers with 6 healthy non-carriers; all these 12 healthy individuals were parents of 6 Q703K positive PFAPA patients.

## Materials and Methods

### Study Population

All children clinically diagnosed with PFAPA (periodic fever, aphthous stomatitis, pharyngitis and cervical adenitis), CAPS (Cryopyrin-associated periodic syndrome) or uAID (undefined autoinflammatory disease) attending the pediatric rheumatology consultation of Western Switzerland at the Lausanne University Hospital and the Geneva University Hospital, from November 2009 to November 2017, had a genetic analysis in the 4 major genes associated with monogenic periodic fevers: *MEFV, TNFRSF1A, MVK, NLRP3*. The data were collected from the retrospective module of the JIR (Juvenile Inflammatory Rheumatism) cohort network. The JIR cohort is an international database which includes patients presenting an inflammatory rheumatism starting in childhood; only the center of Western Switzerland among the JIR network participated in this study. One more patient presenting with recurrent fevers and chronic glomerulonephritis followed in the adult immunology consultation was added in this data collection.

An additional and independent cohort of patients with recurrent fevers was identified from the AID-registry, which is part of the AID-Net (Network for autoinflammatory diseases), a research initiative funded by the German Federal Ministry of Education and Research (BMBF) und supported by the German Rheumatism Research Center (DRFZ) and the German society for pediatric rheumatology (GKJR) (21). Patient data was retrospectively documented between July 2009 and October 2017. All patients with a diagnosis of CAPS or PFAPA and availability of genetic analysis in the four fever genes either provided by the centers or analyzed in Muenster in samples of the Biobank.

Concerning the PFAPA patients, previously published diagnostic criteria (7) were applied. However, because PFAPA is not a well-defined disease and there are no confirmatory tests, the power of these criteria remains limited. New classification criteria, based on a consensus among a large panel of experts and confirmed on a large cohort of PFAPA patients (Eurofever), are currently under investigation and should bring soon some improvements in the classification of PFAPA (8). Consequently patients with disease onset after 5 years of age were included according to the published international multi-center cohort of 301 patients (22). Patients with all three constitutional symptoms were described as having “complete cluster”; otherwise “incomplete cluster” for those with one or two constitutional symptoms. All these children had normal growth and development and did not present any other symptoms suggesting an alternative diagnosis. CAPS diagnosis was suggested by the clinical presentation of patients according to the expert opinion and was confirmed by the new classification criteria (8) and/or genetical analysis.

The symptoms, treatment, response to treatment and family history of the patients with the Q703K *NLRP3* variant have been retrospectively extracted and described from both JIR and AID-Net cohorts. The phenotypes of Q703K-positive and Q703K-negative PFAPA, CAPS and uAID patients were compared, respectively.

In the second part of our study, six pairs of asymptomatic parents of Q703K positive children with PFAPA syndrome were also asked to participate in the study, to compare the inflammasome basal activity between Q703K positive and negative healthy parents.

All subjects received code numbers to guarantee anonymity. Approval for the study was obtained for JIR patients from the Cantonal Ethical Committee in Lausanne and in Geneva and for AID-net patients from the ethics committees and the data protection responsible at the Universities of Duisburg-Essen and Muenster, as well as the Medical Association Nordrhein in Duesseldorf, and the parents/caregivers gave their informed consent according to local ethical regulations.

### Genetic Analysis

Genomic DNA was extracted using standard methods from peripheral mononuclear cells (PBMC). Mutation hotspots in the 4 major genes associated with monogenic periodic fevers were analyzed by using PCR and DNA sequencing. The regions analyzed were as follows: *MEFV* exons 2 and 10 (detects >95% of known pathogenic FMF mutations); *TNFRSF1A* exons 2,3,4, and 6 (detects close to 100% of pathogenic TRAPS mutations); *MVK* exons 9 and 11 (detects ~70% of pathogenic HIDS); and *NLRP3* exon 3 (detects close to 100% of pathogenic CAPS mutations) (23).

### Sample Preparation

Peripheral blood from the subjects was collected in S-Monovette tubes containing EDTA or, for the isolation of sera, clot activators. Anticoagulated blood was collected for DNA extraction and genetic analysis. For cytokine determinations (see below), sera were stored in sterile tubes at −80°C before analysis.

### Monocyte Isolation and Stimulation-Cytokines Measurements

Anticoagulated blood was obtained from the asymptomatic parents of 6 unrelated Q703K-positive children with PFAPA (for each family: father, mother). Peripheral blood mononuclear cells were isolated by Ficoll-Hypaque (Life Technologies). Monocytes were purified by magnetic-associated cell sorting (MACS Monocyte Isolation Kit-Miltenyi). Monocytes were stimulated overnight with ultrapure LPS (200 ng/mL) to activate inflammasome (24). Levels of IL-1β, TNF-a and IL-6 were measured in cell supernatants with ELISA (eBioscience).

### Statistical Analysis

Differences between groups were analyzed by using a two-sample test of proportion with 95% confidence interval, the Wilcoxon signed-rank test, the two-tailed Fisher's exact test, the two-tailed Chi-square with Yates correction and the *t*-test. Statistically significant results are annotated as follows: ^*^P < 0.05, ^**^P < 0.01, ^***^P < 0.001.

## Results

### Association of Q703K Variant With PFAPA, CAPS, and uAID Syndromes

The Q703K variant was observed in 21 out of 150 PFAPA patients (14%, 12/109 in JIR cohort, 9/41 in AID-Net cohort), in 6 out of 24 CAPS patients (25%, 1/8 in JIR cohort, 5/16 in AID-Net cohort) and in 15 out of 36 uAID patients (42%, 7/22 in JIR cohort, 8/11 in AID-Net cohort). All individuals tested positive for Q703K were heterozygous. We compared allele frequencies with data from the Genome Aggregation Database (gnomAD), a publicly available database containing genetic variations from over 10'000 human genomes and exomes, reporting an allelic frequency of 3.8 and 5.1% in general and European populations, respectively. Thus, Q703K showed a significantly increased variant's frequency (*P* < 0.001) in all three PFAPA, CAPS and uAID cohorts, suggesting an association of this variant with the risk of these syndromes (Table 1).

**Table 1:** Q703K allele frequency in our PFAPA, CAPS and uAID patients compared to the healthy population from gnomAD genome and exome data.

**Allele frequency observed in PFAPA (JIR & AID-Net cohorts)**			**Allele frequency observed in CAPS (JIR & AID-Net cohorts)**			**Allele frequency observed in uAID (JIR & AID-Net cohorts)**		
**14% (21/150)**	Allele frequency in total population (gnomAD): 3.8% (10781/280716)	Allele frequency in European population (gnomAD): 5.1% (6584/129092)	**25% (6/24)**	Allele frequency in total population (gnomAD): 3.8% (10781/280716)	Allele frequency in European population (gnomAD): 5.1% (6584/129092)	**42% (15/36)**	Allele frequency in total population (gnomAD): 3.8% (10781/280716)	Allele frequency in European population (gnomAD): 5.1% (6584/129092)
	*P* < 0.001	*P* < 0.001		*P* < 0.001	*P* < 0.001		*P* < 0.001	P < 0.001

### Clinical Characteristics of Patients Carrying the Q703K Variant

We found the *NLRP3* variant Q703K in 42 patients. Of these, 21 were PFAPA patients, 6 had a CAPS phenotype and 15 had an undefined auto-inflammatory disease (uAID). All 21 Q703K-positive PFAPA patients presented with at least one cardinal symptom and 7 had a complete cluster. One patient had neurological symptoms during fever flares (hypotonia, bulging fontanel, loss of contact, seizures). Seven patients had a positive family history of recurrent fever, recurrent pharyngitis or tonsillectomy and one patient had a family history of undefined rheumatic disease. All patients that have been treated with a single steroid dose during the flare responded within 12 h (11/11). Three patients were found to carry also other genetic variants in the *MEFV* gene.

Concerning the 6 Q703K-positive CAPS, 50% of them (3/6) showed a FCAS phenotype and 50% (3/6) a Muckle-Wells phenotype; no patient presented the neonatal form CINCA/NOMID. Two patients were found to carry also other genetic variants in *NLRP3* (D303N, G569A). Of 4 patients with known family history, only 1 was positive. Data for treatment were available in 5 patients; all of them were treated with anti-IL1 agents with a good response.

Clinical data are available in 12 up to 15 Q703K-positive uAID patients. The most frequent symptoms were recurrent fever (10/12), abdominal pain (5/12), oral aphthosis (3/12) and headache (2/12). Of five patients with known family history, two were positive. One patient presented severe proteinuria in the context of chronic glomerulonephritis. Data for treatment were available in 11 patients: 5 of them were treated with anti-IL-1 agents with good response in 4 of them; NSAIDS, steroids and colchicine have been used for the others.

It is important to state that JIR and AID-Net cohorts were assembled before the new classification criteria (8) have been published. For the prupose of our analyses, patients were pothoc re-classified accoring to recently published consensus criteria. Consequently, some patients have been treated with different diagnosis in “real life”; more precisely 12 CAPS patients (10 from AID-Net cohort and 2 from JIR cohort) turned to uAID and 7 CAPS patients (AID-Net cohort) to PFAPA diagnosis for the current analysis. Interestingly, the frequency of the Q703K allele according to the original diagnosis was the following: 11% (16/143) in PFAPA, 44% (19/43) in CAPS, and 3% (7/24) in uAID patients.

### Comparison of Q703K-Positive and Q703K-Negative PFAPA Patients: Similar Phenotype in Both Groups

Comparisons of the clinical characteristics between the Q703K-positive and -negative PFAPA patients were performed in the JIR- and AID-Net cohorts, where all PFAPA patients underwent genetic analysis, and is shown in Table 2. There were no significant differences in the sex ratio, the prevalence of onset after the age of 5 years and the positivity of familial history. One patient in the Q703K-positive group had an atypical presentation with neurological symptoms; no patient had neurological symptoms in the Q703K-negative group. The frequency of the other cardinal symptoms and complete clusters was similar in both groups. Almost all patients in both groups had a good response to steroids.

**Table 2:** Comparison table of clinical characteristics between Q703K-positive vs. -negative PFAPA patients.

	**Q703K+ (n: 21)**	**Q703K- (n: 129)**	***P*-value**
Median age at onset (years.months)	2	1	
Median duration (days)	5^*^	4.25^**^	
Median interval (days)	30^*^	30^**^	
Sex ratio (M/F)	2/1 (14/7)	1/1 (66/63)	0.2402
Positive familial history	8/19	50/102	0.6249
Pharyngitis	17	107/126	0.7450
Oral aphthosis	11	65/120	1
Adenitis	16	78/128	0.2265
Abdominal Pain	13	66/118	0.6420
Complete cluster	7	40/116	1
Onset after 5 y.o.	3	16/127	0.7349
Good response to steroids	11/11 (100%)	101/105 (96%)	1

* *data available in 16 patients*.

** *data available in 127 patients*.

### Comparison of Q703K-Positive and Q703K-Negative CAPS Patients: Higher Prevalence of Pharyngitis in the Q703K-Positive Group; Similar Disease Severity in Both Groups

Comparisons of the clinical characteristics between the Q703K-positive and -negative CAPS patients were performed in both JIR and AID-Net cohorts (Table 3). Phenotypes were quite similar in both groups. Except the higher prevalence of pharyngitis in the Q703K-positive group, there were no statistically significant differences in other clinical characteristics as recurrent fever, urticaria, arthralgia, myalgia, conjunctivitis, adenopathy, abdominal pain, neurologic symptoms, hearing loss. Furthermore, we did not observe significant differences in the disease severity, with the same prevalence of FCAS, MWS and CINCA/NOMID phenotypes in both groups as well as the same frequency of disease complications such as renal insufficiency, ocular complications, growth, and neurodevelopmental delay.

**Table 3:** Comparison table of clinical characteristics between Q703K-positive vs. -negative CAPS patients.

	**Q703K+ (n: 6)**	**Q703K- (n: 20)**	***P*-value**
Median age at onset (years.months)	0.7	0.1	
Median age at diagnosis	6.9	4.1	
Sex ratio (M/F)	2/1	2/1	1
Positive familial history^*^	1/4	9/16	0.5820
Recurrent Fever	3	4	0.2929
Pharyngitis	2	0	0.0462
Abdominal pain	1	2	1
Neurologic symptoms	2	7	1
Adenopathy	1	4	1
Urticaria	4	17	0.5581
Headache	3	2	0.0624
Myalgia	1	2	1
Arthralgia	4	12	1
Rash	1	1	0.4154
Diarrhea	1	0	0.2308
Conjunctivitis	2	5	1
Oral aphthosis	1	0	0.2308
Hearing loss	2	5	1
Disease Complications^**^	1	6	1
FCAS	3	4	0.2929
MWS	3	12	1
CINCA/NOMID	0	4	0.5425

* *familial History is available in only 4 Q703K-positive and 16 negative CAPS patients*.

** *disease complications include ocular complications, renal insufficiency, skeletal and joint deformities, growth and neurodevelopmental delay*.

### Comparison of Q703K-Positive and Q703K-Negative uAID Patients: No Statistically Significant Differences in the Phenotype of Patients

Comparisons of the clinical characteristics between the Q703K-positive and -negative uAID patients with complete datasets were performed in the JIR cohort and AID-Net cohort, where all uAID patients underwent genetic analysis, and is shown in Table 4. No statistically significant differences were found in the prevalence of clinical characteristics, disease complications, nor the positivity of familial history.

**Table 4:** Comparison table of clinical characteristics between Q703K-positive vs. -negative uAID patients.

	**Q703K+ (n: 12)**	**Q703K- (n: 5)**	***P*-value**
Median age at onset (years.months)	2.9	3.8	
Median age at diagnosis	5.3	5.8	
Sex ratio (M/F)	2.3/1	1/4	0.1189
Positive familial history	2/5	2/2	1
Recurrent Fever	10	3	0.5378
Pharyngitis	1	1	0.5147
Abdominal pain	5	2	1
Neurologic symptoms	0	0	1
Adenopathy	1	1	0.5147
Urticaria	1	3	0.0525
Headache	2	1	1
Myalgia	0	1	0.2941
Arthralgia	1	3	0.0525
Rash	1	0	1
Diarrhea	0	0	1
Conjunctivitis	0	0	1
Oral aphthosis	3	2	0.6
Disease Complications	1^*^	0	1

* *severe proteinuria in the context of chronic glomerulonephritis*.

### No Role for Q703K in Monocyte-Derived IL-1β Secretion but Trend in Higher TNF-α Production

**Figure 1:**
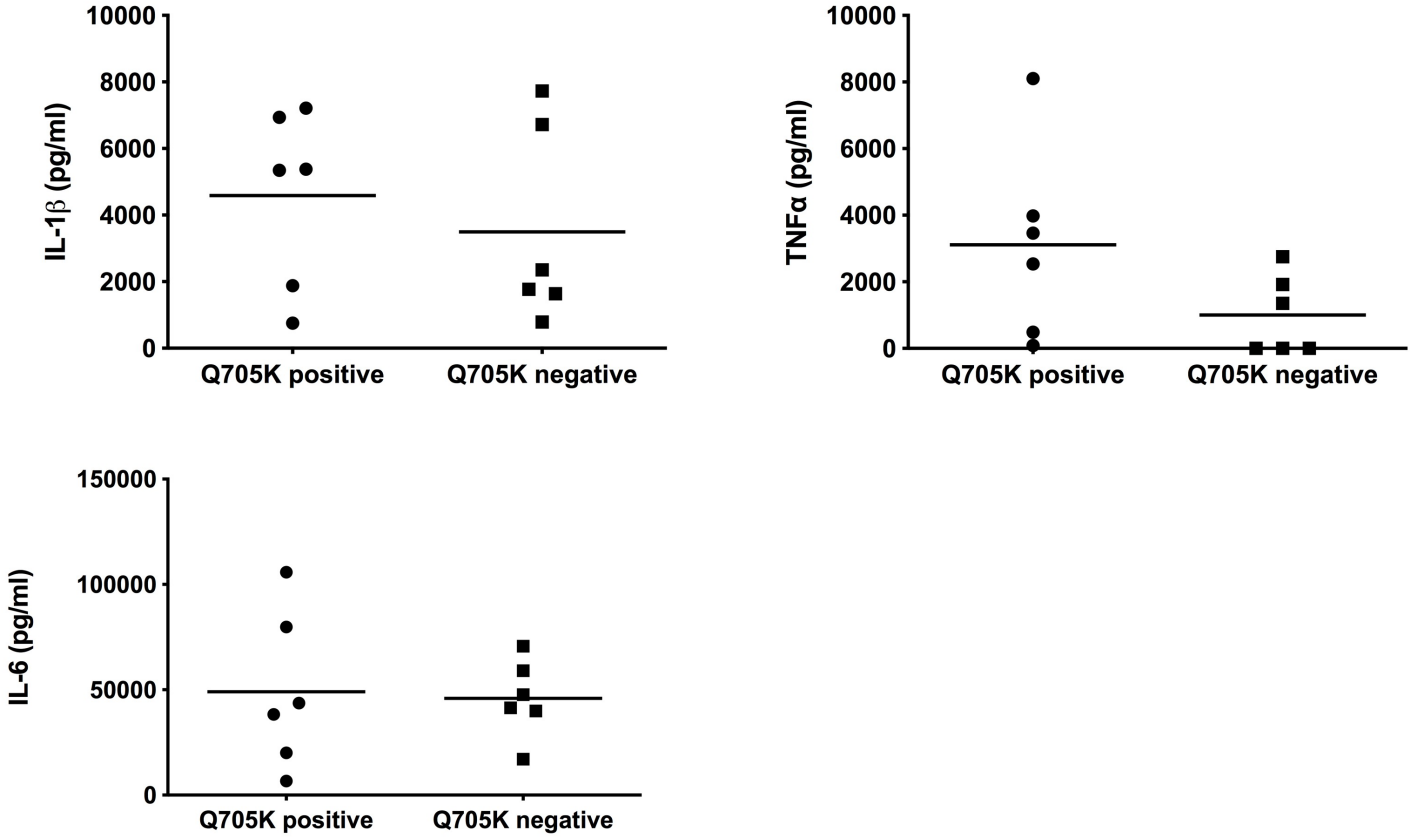
Cytokine secretion by monocytes isolated from PFAPA families. Human PBMC-derived monocytes were cultured in three independent wells and stimulated overnight with 200 ng/ml of LPS. IL1β, TNF-alpha, and IL-6 were measured in supernatants by ELISA. Each dot (mean of values from triplicate monocyte cultures) represents a parent (mother or father) of a PFAPA patient.

We compared the cytokine secretion between 6 Q703K-positive and 6 Q703K-negative healthy parents. As shown in Figure 1, the production of IL-1β, TNF-α or IL-6 were not significantly different between monocytes from Q703K positive and negative asymptomatic parents (Q703K positive: 4583.7 ± 2671.1, 3110 ± 2904.6, and 49043.7 ± 37257.9 pg/ml; Q703K negative: 3499.4 ± 2946.7, 935.6 ± 1259.4, and 45982 ± 18317.4 pg/ml, respectively). However, we observed higher levels of TNF-α production in Q703K-positive healthy parents, even if not significant (3110 ± 2904.6 vs. 935.6 ± 1259.4 pg/ml).

## Discussion

Q703K is an allelic variant of the *NLRP3* gene which pathogenic relevance and penetrance are both poorly understood. Our study supports the assumption of an association of the Q703K variant with a variety of autoinflammatory diseases. In particular, we demonstrate significantly increased variant frequencies in PFAPA, CAPS and uAID cohorts (14, 25, and 42%, respectively), compared to compiled gnomAD data (3–5%), suggesting an association between Q703K and these syndromes (*p* < 0.001).

In recent years, several studies have shown the implication of this variant in autoinflammatory conditions. In particular, Q703K was found in 7% of CAPS patients from the Eurofever Registry (25); Vitale et al. (15) also demonstrated that patients carrying Q703K may present with FCAS-like symptoms, and Insalaco et al. (26) described a new CAPS phenotype associated with the combination of Q703K with a second novel mutation in the *NLRP3* gene. Heterozygosity for Q703K was found in another 17 CAPS cases from Germany: 15 out of 17 cases were CAPS patients and 2 out of 17 were CAPS carriers, 1/17 additionally R92Q TRAPS mutation, 1/17 G569A CINCA variant and 1/17 S52N HIDS variant (27). On the other hand, Naseli et al. (28) found a milder CAPS phenotype in Q703K-positive patients without the typical cytokine pattern observed in typical CAPS patients, suggesting a weak clinical and functional effect of this variant. Furthermore, Kuemmerle-Deschner and her colleagues studied 45 patients with low penetrance *NLRP3* variants (Q703K, V198M, R488K) and found significantly more fever (76%) and gastrointestinal symptoms (73%) compared to CAPS patients with pathogenic *NLRP3* variants. Functional inflammasome testing identified an intermediate phenotype in low-penetrance *NLRP3* variants as compared to wild-type and pathogenetic *NLRP3* variants (20).

Our clinical analyses suggest a possible role of this variant in the clinical inflammatory phenotype of CAPS patients, as they present a statistically significant increased prevalence of pharyngitis. Moreover, it is important to state that Q703K positive CAPS patients have comparable disease severity and complications with those presenting with typical pathogenic *NLRP3* mutations. Interestingly, when analyzing the JIR cohort's PFAPA patients separately, we observe statistically significant increased gastrointestinal symptoms (abdominal pain) in Q703K positive patients compared to the negative ones (Supplementary Table 1), in line with the study of Kuemmerle-Deschner et al. (20). On the other hand, we describe atypical neurologic manifestations in one Q703K PFAPA patient, supporting previous publications that might suggest a tropism of this variant to the central nervous system (29, 30).

Analyzing clinical data most of the Q703K-positive patients in the JIR cohort had a diagnosis of PFAPA, while Q703K-patients from the AID-net cohort had higher rate of CAPS diagnosis. Clinical phenotypes of both diseases are similar, not well-defined because of a number of symptoms typically found in both PFAPA as well as CAPS patients. This difference mirrors the uncertainty of clinicians when laboratories report low-penetrance variants. There is certainly a bias for clinicians to skew a patient's diagnosis into a CAPS-direction if an *NLRP3* variant is detected, and it depends on personal experience and expertise which diagnosis is made. This is also influenced by previous PFAPA-diagnostic criteria that excluded patients with genetic findings in any of the 4 genes responsible for the most common hereditary fever syndromes. A further limitation of our study is the lack of whole exome sequencing in our patient populations, not allowing to exclude modifiers in further genes. Summarizing results of our present cohort study, we suggest considering Q703K *NLRP3* variant as a relevant variant in genetic testing, as it can be associated with PFAPA, CAPS and uAID phenotypes. Moreover, patients carrying this variant probably have a significantly higher risk to develop autoinflammatory disease, but details concerning incidence, severity and prognosis require further studies before this patient group can be integrated into or distinguished from current diagnostic entities. Furthermore, it would be interesting to validate this observation in larger patients' cohorts and in patients with other autoinflammatory diseases classified as FMF or TRAPS to assess whether this finding is specific to PFAPA, CAPS and uAID patients or if it is a more general observation.

In the second part of our study, we aimed to investigate whether Q703K is a gain-of-function variant by comparing the cytokine production between Q703K-positive and Q703K-negative healthy parents. In contrast to the findings of Verma et al. (16, 19) suggesting that Q703K is a gain-of-function alteration leading to an overactive NLRP3 inflammasome, we did not find significant difference in cytokine production between Q703K carriers and non-carriers. However, this is in line with other previous studies (12, 31); in particular Kuemmerle-Deschner et al. showed as well that there was no significant increase of Caspase-1 or Interleukin 1 in Q703K-patients (20). Our hypothesis is that the Q703K variant may be a low-penetrance gain-of-function mutation which might need the presence of further genetic variants to induce auto-inflammation. This is supported by the results of Sahdo et al. (12) showing that carriers of both *NLRP3* Q703K and *CARD8* C10X had higher cytokine levels compared to controls, but carriers of isolated C10X or Q703K variants had similar plasma levels of IL-1β to non-carriers, suggesting a potential synergic effect of different genetic variants in inflammasome activation. Moreover, the combination of Q703K with *CARD8* C10X has been found to correlate with increased caspase-1 activity and IL-1β secretion in patients with CAPS-like symptoms, as well as with dysregulated apoptosis (16, 32). These combined polymorphisms have also been implicated in severe chronic inflammatory diseases, like rheumatoid arthritis and Crohn's disease (17, 18).

In conclusion, the evidence we report in our study supports an association between *NLRP3* Q703K and the risk of autoinflammatory syndromes, as indicated by the significantly increased prevalence of this variant in our PFAPA, CAPS and uAID populations. However, we did not find a functional effect of this mutation as the cytokine secretion was similar in healthy Q703K-positive and -negative individuals. We hypothesize that this mutation might act synergistically with other genetic variants or epigenetic alterations by inducing excessive inflammasome activation and autoinflammation. Lastly, the functional effect of the *NLRP3* Q703K alone is probably not sufficient to induce severe autoinflammatory presentations but contributes to more attenuated phenotypes.

## Key Messages

- The *NLRP3* Q703K variant is associated with various autoinflammatory diseases (PFAPA syndrome, CAPS and undefined AID). However, the correlation between the genotype and the phenotype is unclear and the diagnosis depends on the clinical presentation.
- The effect of Q703K variant remains unclear. However, given its increased prevalence among patients with autoinflammatory diseases, some contribution to the inflammatory phenotype cannot be ruled out.
- The Q703K is not sufficient to induce autoinflammation *per se*, but might be a reduced-penetrance mutation that could have a synergistic effect with other mutations.

## Data Availability Statement

The datasets generated for this study are available on request to the corresponding author.

## Ethics Statement

The studies involving human participants were reviewed and approved by Cantonal Ethical Committee in Lausanne and in Geneva, Ethics committees of Duisburg-Essen and Muenster, as well as the Medical Association Nordrhein in Duesseldorf. Written informed consent to participate in this study was provided by the participants' legal guardian/next of kin.

## Author Contributions

KT, HW, NB, DF, and MH were involved in the conception and design of the study. KT, HW, DF, MH, VH, AV, EL, GHo, J-PH, KP-B, RB, AJ, and GHe organized the databases. KT performed the statistical analysis. NB performed the experiments. MM, IM, and NF performed the genetic studies. KT, HW, EL, NB, DF, and MH analyzed the data. KT wrote the first draft of the manuscript. HW and NB wrote sections of the manuscript. All authors contributed to manuscript revision, read, and approved the submitted version.

## Conflict of Interest

DF has received honoraria from Novartis, Chugai-Roche and SOBI, and he has received research funding from Novartis, Pfizer and SOBI. GHo has received unrestricted Grant for scientific work from Novartis, fees for advisory Board membership from Novartis and Travel cost reimbursement from Swedish orphan. The remaining authors declare that the research was conducted in the absence of any commercial or financial relationships that could be construed as a potential conflict of interest.
