# Supplementary material for: Increased Prevalence of NLRP3 Q703K Variant Among Patients With Autoinflammatory Diseases: An International Multicentric Study
Source: Front Immunol. 2020 May 14;11:877. doi: 10.3389/fimmu.2020.00877 (PMC7241420; doi:10.3389/fimmu.2020.00877)
Supplement: Supplementary file 1 [file Table_1.DOCX]

Supplementary Material

**Supplementary Table 1.** Comparison table of clinical characteristics between Q703K positive vs negative PFAPA patients in the JIR cohorte.

|  | Q703K+ (n : 12) | Q703K- (n :97) | Pvalue |
| --- | --- | --- | --- |
| Median age at onset (years.months) | 01.05 | 01.08 | 0.5773 |
| Sex ratio (M/F) | 2/1 (8/4) | 1.02/1 (49/48) | 0.2906 |
| Median duration (days) | 5 | 4 | 0.3998 |
| Median interval (days) | 30 | 30 | 0.5921 |
| Positive familial history | 8 (67%) | 48 (49%) | 0.2613 |
| Pharyngitis | 11 (92%) | 94 (97%) | 0.3624 |
| Oral aphtosis | 7 (58%) | 52 (54%) | 0.7567 |
| Adenitis | 9 (75%) | 57 (59%) | 0.2776 |
| Abdominal Pain | 10 (83%) | 49 (51%) | 0.0314 |
| Complete cluster | 4 (33%) | 33 (34%) | 0.9622 |
| Onset after 5 y.o. | 2 (17%) | 13 (13%) | 0.7568 |
| Good response to steroids | 9/9 (100%) | 77/80 (96%) | 0.5545 |
